# A Comprehensive Meta-Analysis of the Cardiovascular Adverse Effects of Bispecific Antibody Therapy in Hematologic Malignancies

**DOI:** 10.3390/pathophysiology33030060

**Published:** 2026-08-11

**Authors:** Derek Tai, Navneet Sandhu, Aren Dermarderosian, Norayr Mkrtchyan, Daniel Park, Vinisha Garg, Angel Nguyen, Mojtaba Akhtari

**Affiliations:** 1 Department of Medicine, Loma Linda University Medical Center, Loma Linda, CA 92354, USA; derek77tai@gmail.com; 2 Department of Medicine, University California San Francisco Fresno, Fresno, CA 93701, USA; navneetksandhu1@gmail.com; 3 Department of Chemistry and Biochemistry, University of California Los Angeles, Los Angeles, CA 90095, USA; aren.dermarderosian@gmail.com; 4 School of Medicine, University of California Davis, Sacramento, CA 95817, USA; normkrtchyan@gmail.com; 5 Department of Hematology/Oncology, Harbor-UCLA Medical Center, Torrance, CA 90502, USA; daniel.park@ucsf.edu; 6 Department of Cardiology, Loma Linda University Medical Center, Loma Linda, CA 92354, USA; vgarg@llu.edu; 7 Skaggs School of Pharmacy and Pharmaceutical Science, University of California, La Jolla, San Diego, CA 92093, USA; angelnguyen@ucsd.edu; 8 Division of Transplant, Cellular Therapy and Hematologic Malignancies, Loma Linda University Cancer Center, 11234 Anderson St, Loma Linda, CA 92354, USA

**Keywords:** bispecific antibodies, cardio-oncology, cardiovascular adverse events, cardiotoxicity, malignant hematology

## Abstract

**Introduction:** Bispecific antibodies (BsAbs) are a promising and potent therapeutic intervention for relapsed or refractory (R/R) hematologic malignancies, including acute lymphoblastic leukemia (ALL), multiple myeloma (MM), and B-cell lymphomas. These recombinant molecules are designed to target two distinct antigens or epitopes. While BsAbs have shown significant efficacy in managing hematologic malignancies, toxicities must be carefully monitored and their safety profiles require further investigation. This meta-analysis investigates cardiovascular adverse effects associated with seven BsAbs across multiple clinical trials. **Methods:** A systematic literature review identified seven BsAbs, blinatumomab, elranatamab, epcoritamab, glofitamab, mosunetuzumab, talquetamab, and teclistamab, analyzing 25 clinical trials (Phase I–III) that included monotherapy regimens. The cardiovascular adverse events studied were arrhythmias, heart failure, blood pressure changes, and myocardial infarctions. A meta-analysis using the R meta package calculated proportions and 95% confidence intervals (CIs) and employed a random-effects model to assess heterogeneity. **Results:** Among 3215 patients, pooled analysis revealed significant findings with 13.6% cardiac arrhythmia (95% CI 10.1–18.2%, *p* = 0.05), 13.3% hypotension (95% CI 10–17.3%, *p* < 0.0001), 10.2% tachycardia (95% CI 7.6–13.5%, *p* = 0.0003), and 3.4% sudden death (95% CI 0–80%, *p* = 0.046). Less frequent events were hypertension (8%), acute myocardial infarction (1.5%), heart failure (1.4%), and atrial fibrillation (1.3%). **Discussion:** As BsAbs see increased use in R/R hematologic malignancies, cardiovascular complications must be closely monitored. This analysis highlights hypotension, cardiac arrhythmias, tachycardia, and sudden death as significant adverse effects. Although study heterogeneity and limited patient-level data may have influenced incidence estimates, these findings support routine cardiovascular monitoring and underscore the need for prospective studies to identify high-risk patients and optimize prevention and management strategies. Oncologists, cardiologists, and pharmacists should establish strategies for early detection and management to optimize patients’ outcomes.

## 1. Introduction

Bispecific antibodies (BsAbs) are a promising and potent therapeutic intervention for relapsed or refractory (R/R) hematologic malignancies, including acute lymphoblastic leukemia (ALL), multiple myeloma (MM), and B-cell lymphomas. Typical monoclonal antibodies target one epitope, but these recombinant molecules are designed to target two distinct antigens or epitopes that allow them to target immune and tumor cells concurrently. This mechanism of action allows them to have synergistic effects. Besides hematologic malignancies, they have a wide range of clinical applications including vascular, ocular, and infectious conditions. The first FDA-approved BsAb was blinatumomab in 2014 [1]. Since then, there have been 11 additional FDA-approved BsAbs, with over 100 in development.

Blinatumomab targets CD19 on B-cells and CD3 on T-cells and is approved for the treatment of CD19-positive B-cell precursor ALL in various stages, including minimal residual disease (MRD)-positive and R/R disease [1,2,3,4,5,6,7,8,9,10,11,12,13]. Elranatamab targets B-cell maturation antigen (BCMA) and CD3, and is used in R/R multiple myeloma [14,15,16,17,18,19,20]. Epcoritamab and Glofitamab target CD20 on B-cells and CD3 on T-cells and are approved for use in R/R diffuse large B-cell lymphoma (DLBCL) and have shown efficacy in heavily pretreated patients, including those who have failed CAR T-cell therapy [21,22,23,24,25,26,27,28,29,30,31,32,33,34,35,36,37,38,39,40,41,42,43,44,45,46,47,48,49,50,51]. Mosunetuzumab also targets CD20 and CD3 and is approved for R/R follicular lymphoma, demonstrating promising response rates and durability in third-line and beyond [52,53,54,55,56,57,58,59,60,61,62,63,64,65,66,67,68,69,70]. Talquetamab targets G-protein coupled receptor family C group 5 member D (GPCR5D) and CD3 and is used in MM, particularly in patients who have exhausted other treatment options [71,72,73,74,75,76,77]. Teclistamab targets BCMA and CD3 and is used in R/R MM. The National Comprehensive Cancer Network (NCCN) guidelines highlight its efficacy in patients with triple-class refractory disease [78,79,80,81,82,83,84,85].

While BsAbs have shown significant efficacy in managing hematologic malignancies, toxicities must be carefully monitored and their safety profiles require further investigation. BsAb induced T-cell depletion, neutropenia, and hypogammaglobulinemia are known to cause an increased risk of bacterial, viral, and opportunistic infections. Each BsAb has a different target as seen with CD19 in blinatumomab, CD20 in epcoritamab, glofitamab, and mosunetuzumab, BCMA in elranatamab and teclistamab, and GPRC5D in talquetamab. Despite each BsAb having different targets, they all cause direct T-cell activation and on-target off-tumor toxicities through a shared mechanism of engaging T-cells through CD3, contributing to the higher risk of side effects [86,87]. Notably, cardiovascular adverse effects have not been extensively studied despite reported incidents in agents such as blinatumomab and teclistamab. This meta-analysis aims to investigate the cardiovascular toxicities associated with seven BsAbs across multiple clinical trials and studies in hematologic malignancies.

## 2. Methods

### 2.1. Literature Search Methods

A comprehensive literature search was conducted using Medline (PubMed) and Embase to identify studies reporting cardiovascular adverse events associated with BsAb therapy in the context of hematologic malignancies. Search keywords and MeSH terms included “bispecific antibodies,” “cardiotoxicity,” “cardiac adverse events,” and “hematologic malignancies,” as well as these terms in conjunction with the specific drugs relevant to this study: “blinatumomab,” “elranatamab,” “epcoritamab,” “glofitamab,” “mosunetuzumab,” “talquetamab,” and “teclistamab.” Only peer-reviewed studies published in English dating back to 2014 at the earliest were included. Conference abstracts were also screened using the ClinicalTrials.gov website, and proceedings from the American Society of Hematology (ASH) and the European Hematology Association (EHA) were analyzed.

Inclusion criteria consisted of randomized controlled trials (RCTs) that evaluated BsAb monotherapies, reported cardiotoxic adverse effects, and provided sufficient relevant data for inclusion in the meta-analysis. Exclusion criteria encompassed preclinical studies, case reports, and, notably, studies involving combination therapies.

The final dataset in this study included 25 clinical trials spanning various phases:

Phase I (*n* = 5): MagnetisMM-1(NCT03269136); MagnetisMM-2 (NCT04798586); A Phase 1b Open-Label Study Investigating the Safety and Pharmacokinetics of Administration of Subcutaneous Blinatumomab for the treatment of Relapsed/Refractory Indolent Non-Hodgkin’s Lymphoma (NCT02961881); Safety Study of the Bispecific T-cell Engager Blinatumomab (MT103) in Patients With Relapsed NHL (NCT00274742); A Study to Evaluate Glofitamab as Single Agent Administered After Pretreatment With Obinutuzumab in Chinese Patients With Relapsed/Refractory Diffuse Large B-Cell Lymphoma (NCT04657302).

Phase I/II (*n* = 8): MonumenTAL-1(NCT03399799) (NCT04634552); Study of Blinatumomab in Japanese Patients With Relapsed/Refractory B-precursor Acute Lymphoblastic Leukemia (NCT02412306); MajesTEC-1 (NCT03145181 and NCT04557098); First-in-Human (FIH) Trial in Patients With Relapsed, Progressive or Refractory B-Cell Lymphoma (EPCORE™ NHL-1) (NCT03625037); A Dose Escalation Study of Glofitamab (RO7082859) as a Single Agent and in Combination With Obinutuzumab, Administered After a Fixed, Single Pre-treatment Dose of Obinutuzumab in Participants With Relapsed/Refractory B-cell Non-hodgkin’s Lymphoma (NCT03075696); A Safety, Efficacy and Pharmacokinetic Study of BTCT4465A (Mosunetuzumab) as a Single Agent and Combined With Atezolizumab in Non-Hodgkin’s Lymphoma (NHL) and Chronic Lymphocytic Leukemia (CLL) (NCT02500407); A Phase Ib/II Study Investigating the Safety, Tolerability, Pharmacokinetics, and Efficacy of Mosunetuzumab (BTCT4465A) in Combination With CHOP or CHP-Polatuzumab Vedotin in Participants With B-Cell Non-Hodgkin Lymphoma (NCT03677141); A Study to Evaluate the Safety and Efficacy of Mosunetuzumab (BTCT4465A) in Combination With Polatuzumab Vedotin in B-Cell Non-Hodgkin Lymphoma (NCT03671018).

Phase II (*n* = 9): MagnetisMM-3 (NCT04649359); Safety and Efficacy of Blinatumomab in Adults With Newly Diagnosed High-risk Diffuse Large B-Cell Lymphoma (NCT03023878); Blinatumomab Maintenance Following Allogeneic Hematopoietic Cell Transplantation for Patients With Acute Lymphoblastic Leukemia (NCT02807883); Clinical Study With Blinatumomab in Patients With Relapsed/Refractory Diffuse Large B-cell Lymphoma (DLBCL) (NCT01741792); Blinatumomab in Adults With Relapsed/Refractory Philadelphia Positive B-precursor Acute Lymphoblastic Leukemia (NCT02000427); Confirmatory Phase II Study of Blinatumomab (MT103) in Patients With Minimal Residual Disease of B-precursor Acute Lymphoblastic Leukemia (NCT01207388); Study of the BiTE^®^ Blinatumomab (MT103) in Adult Patients With Relapsed/Refractory B-Precursor Acute Lymphoblastic Leukemia (ALL) (NCT01209286); Clinical Study With Blinatumomab in Patients With Relapsed/Refractory B-precursor Acute Lymphoblastic Leukemia (ALL) (NCT01466179); Phase II Study of the BiTE^®^ Blinatumomab (MT103) in Patients With Minimal Residual Disease of B-precursor Acute Lymphoblastic Leukemia (ALL) (NCT00560794).

Phase II/III (*n* = 1): Study to Evaluate Safety and Efficacy of Blinatumomab in Subjects With Relapsed/Refractory (R/R) Aggressive B-Cell NHL (NCT02910063).

Phase III (*n* = 2): TOWER study (NCT02013167); Efficacy and Safety of the BiTE Antibody Blinatumomab in Chinese Adult Subjects With Relapsed/Refractory B-precursor Acute Lymphoblastic Leukemia (ALL) (NCT03476239).

Data extraction began with reviewers screening titles and abstracts for eligibility, followed by a full-text review. Extracted data included trials (including phases), total population, average survival time after intervention, age, gender, prior therapies, and most importantly, cardiology-related adverse events. Definitions for these events were standardized across studies to minimize heterogeneity.

### 2.2. Outcome Measures

Cardiovascular adverse effects included atrial fibrillation, ventricular fibrillation, atrial flutter, acute myocardial infarction, sudden death, cardiac arrest, cardiorespiratory arrest, cardiopulmonary failure, tachyarrhythmia, bradyarrhythmia, dysrhythmia, bleeding, hypotension, orthostatic hypotension, hypertension, thromboembolic events, hemorrhage, angina pectoris, cardiac-related chest pain, cardiogenic shock, heart failure, pericardial effusion, cardiovascular insufficiency, supraventricular extrasystoles, ventricular tachycardia, palpitations, cough, dyspnea, hypoxia, acute respiratory failure, respiratory failure, respiratory arrest, pulmonary hemorrhage, capillary leak syndrome, hematoma, intra-abdominal hematoma, embolism, phlebitis, femoral artery occlusion, subclavian vein thrombosis, vascular collapse, hypovolemic shock, venous thrombosis, thrombophlebitis, thrombosis, aortic occlusion, vena cava thrombosis, deep vein thrombosis, and pulmonary embolism.

### 2.3. Statistical Methods

**Figure 1:**
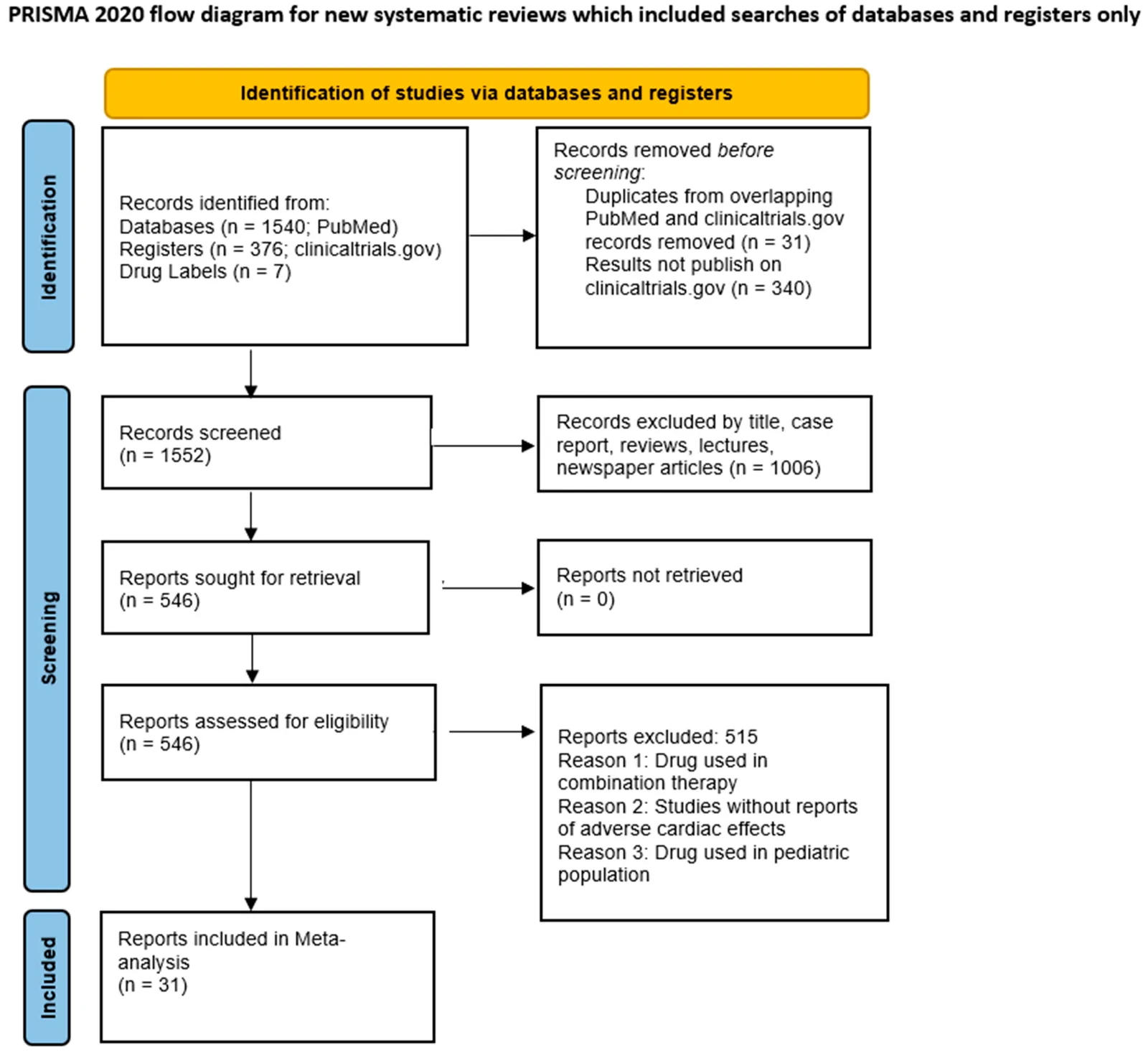
A PRISMA flow diagram of studies screened for meta-analysis. Detailed reasons for exclusion were recorded during screening but category-specific counts were not retained after completion of the review.

Meta-analyses were performed using the R statistical software package (version 4.3.2) with the meta and metafor packages. Pooled event rates, along with their corresponding 95% confidence intervals, were calculated using a random-effects model to account for variability across studies. Heterogeneity was quantified using the I^2^ statistic, with values interpreted as follows: 0–25% (low heterogeneity), 26–50% (moderate heterogeneity), and greater than 50% (high heterogeneity). Further subgroup analyses were conducted based on BsAb type. Excluding studies with small sample sizes and high risks of bias helped test the robustness of findings, and cumulative meta-analysis was conducted to examine the effect. The Preferred Reporting Items for Systematic Reviews and Meta-Analyses (PRISMA) guidelines were followed throughout the study. A PRISMA flow diagram detailing the study selection process is included in Figure 1. In addition to adherence to the PRISMA 2020 reporting guidelines, the methodological quality of this systematic review was evaluated using the AMSTAR 2 (A MeaSurement Tool to Assess Systematic Reviews 2) framework [88]. A completed AMSTAR 2 checklist is provided in the Supplementary Materials.

## 3. Results

### 3.1. Eligible Studies and Characteristics

A total of 25 studies and trials were chosen for the meta-analysis and the most recent data were used for studies or trials providing updated data. The data were collected from a total of 31 district publications reporting on the 25 clinical trials. Ultimately, 31 publications were utilized for analysis as 6 additional publications added patient cohorts to the ongoing trials without overlapping on prior patient cohorts. In total, 3215 patients were included across 31 publications. Table 1 describes the baseline characteristics of the patients in the studies. The patient population comprised individuals with various hematologic malignancies, including MM, B-ALL, DLBCL, and non-Hodgkin lymphomas (NHLs). The median age of included patients was 64 years, with a range of 28 to 75 years. Of 3215 patients, 1710 were male and 1161 were female, the remaining 344 patients were not documented as male or female. The number of prior treatments varied widely, with many patients having received multiple lines of therapy before BsAb administration. The median number of prior therapies before starting a BsAb was 3. Detailed prior treatment regimens were inconsistently reported across the included studies. Most publications reported only the median number of prior treatment lines, precluding a systematic comparison of specific prior therapies or cumulative exposure to cardiotoxic agents. The median percentage of patients’ Eastern Cooperative Oncology Group (ECOG) performance status at 1 or 0 was 96%.

### 3.2. Evaluation of Bispecific Antibody Cardiovascular Adverse Events

Individual BsAbs were analyzed for cardiovascular adverse events. Of the 7 BsAbs, blinatumomab was the most represented.

Blinatumomab was associated with 11% hypotension (95% CI 9.3–13.1%, *p* = 0.74, I^2^ = 0%), 10.4% tachycardia (95% CI 6–17.5%, *p* = 0.12, I^2^ = 63.3%), 8.1% hypertension (95% CI 5.2–12.5%, *p* = 0.088, I^2^ = 43.6%), 7.6% cardiac arrhythmia (95% CI 1.3–33.8%, *p* = 0.029, I^2^ = 62.8%), 2.3% bradycardia (95% CI 0.3–16.1%, *p* = 0.91, I^2^ = 53.6%), 2% supraventricular tachycardia (95% CI 0–5.3%, *p* = 0.087, I^2^ = 59.1%), 1.7% heart failure (95% CI 0.3–9.7%, *p* = 0.079, I^2^ = 52.1%), 1.5% atrial fibrillation (95% CI 0.2–1.3%, *p* = 0.029, I^2^ = 66.7%), 0.6% cardiac arrest (95% CI 0–4.9%, *p* = 0.57, I^2^ = 0%), and 0.5% cardiopulmonary failure (95% CI 0–46.1%, *p* = 0.58, I^2^ = 0%). Significant findings included atrial fibrillation (*p* = 0.029) and cardiac arrhythmia (*p* = 0.029).

Mosunetuzumab was associated with 13.6% hypotension (95% CI 3.4–41.8%, *p* < 0.0001, I^2^ = 90.7%), 8.1% tachycardia (95% CI 2–28.2%, *p* = 0.036, I^2^ = 70%), and 0.8% acute myocardial infarction (95% CI 0.2–2.4%, *p* = 0.8, I^2^ = 0%). Significant findings included tachycardia (*p* = 0.036) and hypotension (*p* < 0.0001).

Glofitamab was associated with 1.4% acute myocardial infarction (95% CI 0–99.9%, *p* = 0.22, I^2^ = 34.8%).

Elranatamab was associated with 9.1% hypotension (95% CI 2.4–29.3%, *p* = 0.66, I^2^ = 0%), 8% hypertension (95% CI 6.3–10.2%, *p* = 0.94, I^2^ = 0%), 6.9% sudden death (95% CI 0–100%, *p* = 0.059, I^2^ = 71.8%), and 6.5% tachycardia (95% CI 0.8–36.7%, *p* = 0.58, I^2^ = 0%).

### 3.3. Evaluation of Pooled Cardiovascular Adverse Events

Table 2 provides a summary of the pooled analysis for the BsAbs. Forest plots were completed and provided a pooled analysis of hypotension, cardiac arrhythmias, tachycardia, hypertension, sudden death, acute myocardial infarction, heart failure, and atrial fibrillation. Significant forest plots in hypotension, cardiac arrhythmia, tachycardia, and sudden death are shown in Figure 2, Figure 3, Figure 4 and Figure 5. The remaining forest plots for additional subgroup analyses are provided in the Supplementary Data Section (Figures S1–S29).

**Figure 2:**
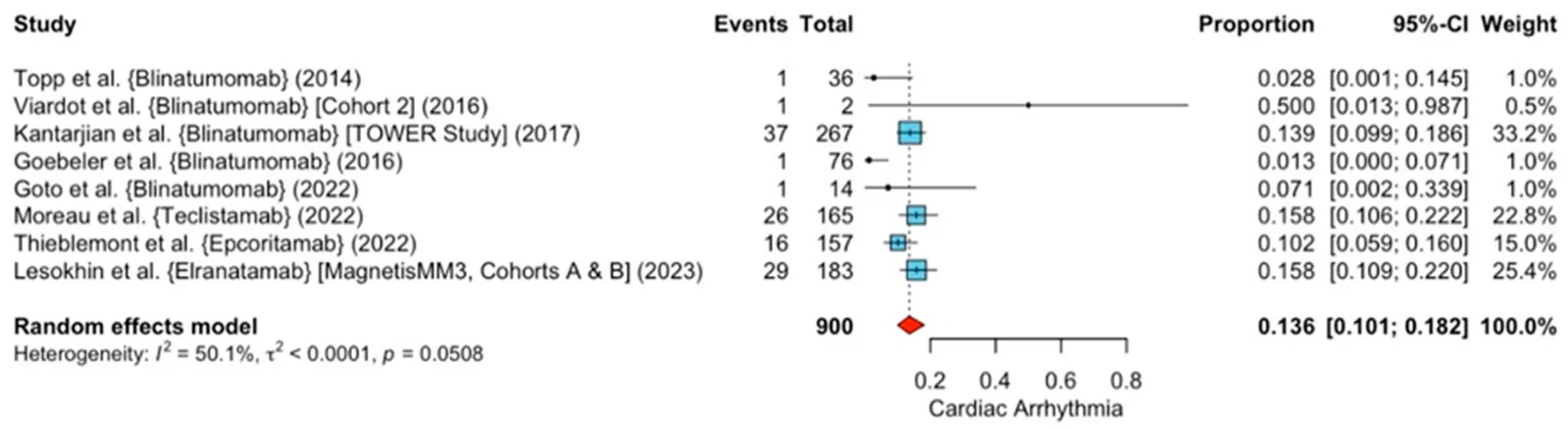
Forest plot of cardiac arrhythmia for blinatumomab, teclistamab, epcoritamab, and elranatamab. Blue squares represent the estimated cardiac arrhythmia rate from each individual study, with larger squares indicating studies that contributed more weight to the analysis. Horizontal lines represent uncertainty ranges (95% confidence intervals). The red diamond represents the overall pooled estimate across all studies, with its width reflecting uncertainty around the combined result.

Pooled analysis of blinatumomab, teclistamab, epcoritamab, and elranatamab was associated with 13.6% cardiac arrhythmia (95% CI 10.1–18.2%, *p* = 0.05, I^2^ = 50.1%), as seen in Figure 2.

**Figure 3:**
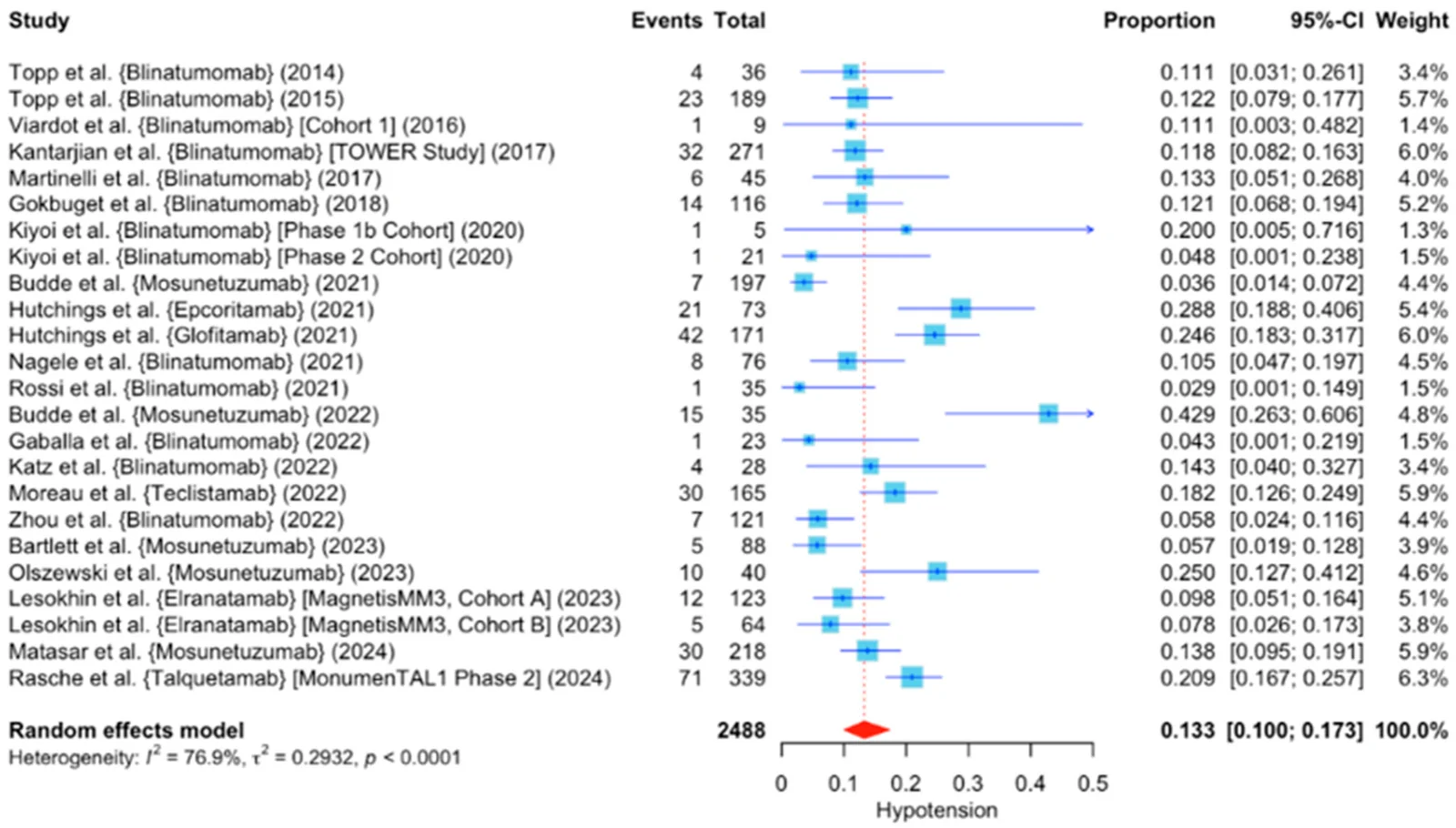
Forest plot of hypotension for blinatumomab, mosunetuzumab, epcoritamab, glofitamab, teclistamab, elranatamab, and talquetamab. Blue squares represent the estimated hypotension rate from each individual study, with larger squares indicating studies that contributed more weight to the analysis. Horizontal lines represent uncertainty ranges (95% confidence intervals). The red diamond represents the overall pooled estimate across all studies, with its width reflecting uncertainty around the combined result.

Pooled analysis of blinatumomab, mosunetuzumab, epcoritamab, glofitamab, teclistamab, elranatamab, and talquetamab was associated with 13.3% hypotension (95% CI 10–17.3%, *p* < 0.0001, I^2^ = 76.9%), as seen in Figure 3.

**Figure 4:**
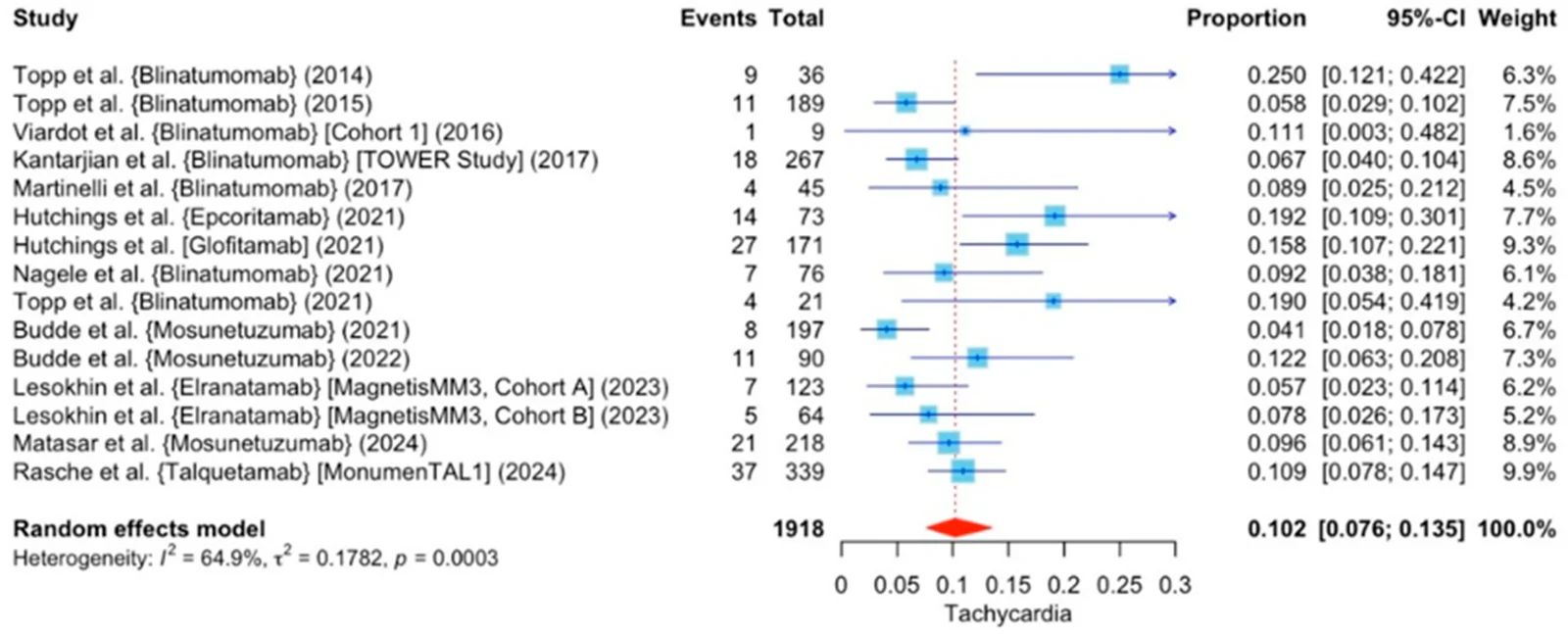
Forest plot of tachycardia for blinatumomab, epcoritamab, glofitamab, mosunetuzumab, elranatamab, and talquetamab. Blue squares represent the estimated tachycardia rate from each individual study, with larger squares indicating studies that contributed more weight to the analysis. Horizontal lines represent uncertainty ranges (95% confidence intervals). The red diamond represents the overall pooled estimate across all studies, with its width reflecting uncertainty around the combined result.

Pooled analysis of blinatumomab, epcoritamab, glofitamab, mosunetuzumab, elranatamab, and talquetamab was associated with 10.2% tachycardia (95% CI 7.6–13.5%, *p* = 0.0003, I^2^ = 64.9%), as seen in Figure 4.

Pooled analysis of blinatumomab and elranatamab was associated with 8% hypertension (95% CI 5.8–11%, *p* = 0.19, I^2^ = 27.5%).

**Figure 5:**
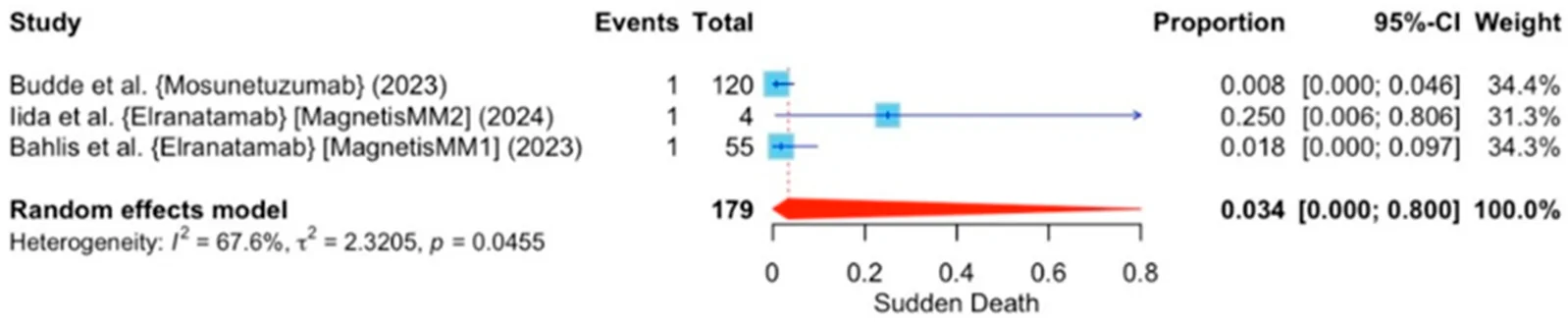
Forest plot of sudden death for mosunetuzumab and elranatamab. Blue squares represent the estimated sudden death rate from each individual study, with larger squares indicating studies that contributed more weight to the analysis. Horizontal lines represent uncertainty ranges (95% confidence intervals). The red diamond represents the overall pooled estimate across all studies, with its width reflecting uncertainty around the combined result.

Pooled analysis of mosunetuzumab and elranatamab was associated with 3.4% sudden death (95% CI 0–80%, *p* = 0.046, I^2^ = 67.6%), as seen in Figure 5.

Pooled analysis of blinatumomab, glofitamab, and mosunetuzumab was associated with 1.5% acute myocardial infarction (95% CI 0.7–3.1%, *p* = 0.5, I^2^ = 0%).

Pooled analysis of blinatumomab and epcoritamab was associated with 1.4% heart failure (95% CI 0.3–5.8%, *p* = 0.085, I^2^ = 48.3%).

Pooled analysis of blinatumomab, mosunetuzumab, and elranatamab was associated with 1.3% atrial fibrillation (95% CI 0.4–4.4%, *p* = 0.06, I^2^ = 52.5%).

Among the pooled analysis of BsAbs, significant findings included hypotension (*p* < 0.0001), cardiac arrhythmia (*p* = 0.05), tachycardia (*p* = 0.0003) and sudden death (*p* = 0.046).

## 4. Discussion

Research to date has shown the efficacy of BsAbs in the management of R/R hematologic malignancies including ALL, MM, and B-NHL such as DLBCL and follicular lymphoma; however, cardiac adverse effects have not been thoroughly studied. This meta-analysis was conducted to compile cardiac adverse events amongst approved BsAbs.

Our meta-analysis, with 3215 patients, provides a comprehensive analysis of the cardiovascular toxicities associated with BsAbs used in hematologic malignancies. The findings demonstrate a variety of cardiovascular risks across blinatumomab, elranatamab, epcoritamab, glofitamab, mosunetuzumab, talquetamab, and teclistamab, highlighting the need for close cardiac monitoring in patients on BsAbs.

### 4.1. Evaluation of Independent Bispecific Antibodies

Blinatumomab was the most represented BsAb in this analysis and was significant in atrial fibrillation (1.5%, *p* = 0.029) and cardiac arrhythmia (7.6%, *p* = 0.029). Additionally, cardiac arrest, supraventricular tachycardia, tachycardia, bradycardia, hypertension, hypotension, heart failure, and cardiopulmonary failure were observed but without statistical significance. Given its frequent use in ALL, patients receiving blinatumomab should undergo baseline cardiovascular assessments with an electrocardiogram, and practicing hematologists and oncologists should remain attentive for arrhythmic complications.

Mosunetuzumab was significantly associated with tachycardia (8.1%, *p* = 0.036) and hypotension (13.6%, *p* < 0.0001). Acute myocardial infarctions were documented but did not reach statistical significance. Regardless, these findings suggest a need for hemodynamic and cardiac rhythm monitoring, particularly in patients with cardiac comorbidities. While acute myocardial infarction was not statistically significant, the long-term cardiovascular impact should still be monitored with use of mosunetuzumab.

Glofitamab was associated with a 1.4% incidence of acute myocardial infarction but it was not statistically significant (*p* = 0.22). While reassuring, the wide confidence interval suggests variability, demonstrating a need for additional studies and longitudinal data to better characterize glofitamab’s cardiovascular toxicities.

Elranatamab was associated with tachycardia (6.5%), hypotension (9.1%), hypertension (8%), although none approached statistical significance. There was a 6.9% incidence of sudden death, approaching statistical significance (*p* = 0.059). Given these concerning findings, particularly for sudden death, patients should be closely monitored and risk-stratified when receiving elranatamab to minimize severe adverse events.

There were not enough reported data to analyze cardiovascular adverse events for epcoritamab, talquetamab, and teclistamab independently.

### 4.2. Evaluation of Pooled Bispecific Antibodies

The pooled analysis further revealed significant findings for tachycardia (*p* = 0.0003), cardiac arrhythmia (*p* = 0.05), hypotension (*p* < 0.0001), and sudden death (*p* = 0.046). The high heterogeneity (I^2^ > 50%) seen in several cardiovascular toxicities, including atrial fibrillation (52.5%), tachycardia (64.9%), and sudden death (67.6%), suggests variability in patient populations, comorbidities, and study methodologies although these were not documented in the clinical trials. While BsAbs are an important advancement in malignant hematology therapies, their cardiovascular toxicity risks should not be overlooked, particularly in patients with pre-existing cardiac disease. Many of these clinical studies did not account for pre-existing heart diseases but future studies can investigate and stratify risks in patients on BsAbs with and without pre-existing cardiac diseases.

Another important consideration is prior exposure to cardiotoxic therapies before BsAb treatment. Patients with relapsed or refractory hematologic malignancies frequently receive multiple prior lines of therapy, including potentially cardiotoxic therapeis such as anthracycline-containing chemotherapy for aggressive B-cell lymphomas, proteasome inhibitors and immunomodulatory agents for MM, hematopoietic stem cell transplantation, CAR-T-cell therapy, and, in selected patients, mediastinal radiation. These prior treatments may contribute to cumulative cardiovascular injury and increase susceptibility to subsequent cardiac adverse events. Unfortunately, the included clinical trials generally reported only the number of previous treatment lines rather than detailed treatment histories, cumulative anthracycline doses, or baseline cardiac assessments. Consequently, this meta-analysis could not determine whether cardiovascular events were attributable to BsAb therapy alone or reflected cumulative toxicity from prior cancer-directed therapies. Future prospective studies should systematically collect these variables to better define independent predictors of BsAb-associated cardiotoxicity.

The significant association in the pooled analysis of mosunetuzumab and elranatamab with sudden death (3.4%, *p* = 0.046) is cause for concern. While the wide confidence interval suggests the need for further research, this finding encourages diligent post-marketing surveillance and prospective studies to identify not only high-risk patients and predisposing factors but also preventative measures.

### 4.3. Clinical Implications

A key finding in our analysis is the high incidence of hypotension and tachycardia across multiple studies. These events may be attributed to immune-mediated inflammatory responses such as cytokine release syndrome or direct drug effects on autonomic regulation [90]. The presence of atrial fibrillation, albeit at a lower frequency, raises concerns about potential arrhythmogenic effects, particularly in patients with predisposing factors such as hypertension or structural heart disease. These findings should be interpreted within the context of heavily pretreated patient populations, many of whom may have accumulated cardiovascular risk from prior therapies such as anthracycline-containing chemotherapy or hematopoietic stem cell transplantation, emphasizing the importance of individualized cardiovascular risk assessment before initiation of BsAb therapy.

The clinical implication from both the individual and pooled BsAb cardiotoxicity findings emphasize a need for baseline cardiovascular assessments prior to initiating BsAb therapy. Establishing baseline cardiac rhythms through electrocardiograms and monitoring hemodynamic vitals would be beneficial, especially for patients receiving blinatumomab, mosunetuzumab, and elranatamab, which were associated with significant cardiac events. Early intervention and medical management, including the use or control of rate-controlling medications or antihypertensives, may be warranted in high-risk individuals.

Given the growing utilization of BsAbs in malignant hematologic disorders, future studies should focus on identifying specific patient populations at increased risk of developing cardiovascular toxicities as well as creating management strategies in order to reduce adverse events. Prospective trials may consider standardized cardiovascular monitoring protocols to ensure patient safety while maximizing therapeutic efficacy.

In conclusion, while BsAbs offer significant promise in the treatment of hematologic malignancies, their cardiovascular toxicities must also be carefully considered. Practicing oncologists and hematologists, cardiologists, and pharmacists should take care to be proactive in monitoring their patients and provide early interventions when signs of cardiotoxicities are observed while on BsAbs to optimize treatment outcomes.

## 5. Future Directions

Future research should focus on mechanistic studies to better understand the underlying causes of BsAb-induced cardiotoxicity and prospective clinical trials and post-marketing monitoring to evaluate preventive strategies, such as using beta-blockers, antihypertensives, hemodynamic monitoring, and cardiac rhythm monitoring. Understanding the impact of individual BsAbs will be crucial in tailoring treatment options and optimizing patient outcomes.

## 6. Limitations

Despite the robust sample size, this meta-analysis has several limitations to be considered. First, the included studies exhibited varying degrees of heterogeneity, which may have influenced the overall estimates of cardiovascular toxicity. Differences in patient populations, study designs, and reporting standards may have contributed to these variations. Second, the retrospective nature of some included studies may introduce selection bias and limit the ability to establish causality. Third, the limited documentation and lack of individual patient data may have resulted in underreported cardiac adverse events. Finally, variations in BsAb protocols and supportive care measures across studies may have affected the reported incidence of cardiac adverse events. Furthermore, most included studies did not provide detailed information regarding prior treatment regimens, cumulative anthracycline exposure, radiation therapy, baseline cardiac function, or pre-existing cardiovascular disease, limiting our ability to evaluate the contribution of prior cardiotoxic therapies to subsequent cardiovascular adverse events observed during BsAb treatment. Future prospective studies with standardized reporting and larger patient cohorts are necessary to validate and expand upon these findings.

## 7. Conclusions

This meta-analysis highlights the cardiovascular risks associated with BsAbs in patients with hematologic malignancies. As BsAbs have demonstrated promising efficacy and their use is being increased in the treatment of R/R hematologic malignancies, a wide variety of cardiac toxicities have been observed in these patients and more data on these toxicities have been documented. This meta-analysis has identified tachycardia, cardiac arrhythmias, and hypotension as the most significant cardiac adverse events in a pooled analysis of multiple BsAbs. While myocardial infarction, atrial fibrillation, hypertension, heart failure, and sudden death were observed at lower rates, their potential impact warrants further investigation. Given the increasing use of BsAbs, future research should focus on identifying patients at risk of cardiac adverse events, optimizing supportive care, and refining treatment guidelines in order to mitigate cardiovascular toxicity while preserving therapeutic efficacy. Practicing oncologists and hematologists, cardiologists, and pharmacists need not only to be aware of these potential toxicities, but also to establish strategies for cardiac monitoring, prevention, and management to provide quality care to cancer patients.

## Figures and Tables

**Table 1:** Baseline characteristics of studies.

Study	Drug	Pathology	Number of Patients	Median Age (Years)	Number of Prior Treatments (Median)	ECOG Performance Status ≤ 1 (%)
Lesokhin et al. [MagnetisMM-3, Cohort A] (2023) [14]	Elranatamab	MM	123	68	5	94.3
Lesokhin et al. [MagnetisMM-3, Cohort B] (2023) [14]	Elranatamab	MM	64 *	65.7 *	7.5 *	-
Lesokhin et al. [MagnetisMM-3, Cohorts A &B] (2023) [14]	Elranatamab	MM	187 **	68 **	-	-
Bahlis et al. [MagnetisMM-1] (2023) [15]	Elranatamab	MM	55	64	5	90.9
Iida et al. [MagnetisMM-2] (2024) [19]	Elranatamab	MM	4	68.5	7.5	100
Rasche et al. [MonumenTAL-1 Phase 2] (2024) [73]	Talquetamab	MM	339 **	67	5	-
Kantarjian et al. [TOWER Study] (2017) [1]	Blinatumomab	B-ALL	271	40.8	-	84.8
Katz et al. (2022) [89]	Blinatumomab	High-risk DLBCL	28	64	-	-
Gaballa et al. (2022) [90]	Blinatumomab	ALL Post-Allogenic HSCT	23 *	29	-	-
Rossi et al. (2021) [91]	Blinatumomab	R/R Indolent NHL	35 *	64	-	-
Zhou et al. (2022) [9]	Blinatumomab	B-ALL	121 *	35.4 *	-	80
Viardot et al. [Cohort 1] (2016) [92]	Blinatumomab	DLBCL	9	71.8 *	3	-
Viardot et al. [Cohort 2] (2016) [92]	Blinatumomab	DLBCL	2	64.5 *	2	-
Viardot et al. [Cohort 3] (2016) [92]	Blinatumomab	DLBCL	14	57.1	3	-
Martinelli et al. (2017) [4]	Blinatumomab	B-ALL	45	55	-	80
Kiyoi et al. [Phase 1b Cohort] (2020) [11]	Blinatumomab	R/R B-ALL	5	58	-	80
Kiyoi et al. [Phase 2 Cohort] (2020) [11]	Blinatumomab	R/R B-ALL	21	43	-	95
Goto et al. (2022) [93]	Blinatumomab	R/R B-ALL	14	-	-	-
Coyle et al. (2020) [94]	Blinatumomab	R/R Aggressive B-cell Non-Hodgkin Lymphoma	41	56	-	90
Gokbuget et al. (2018) [2]	Blinatumomab	B-ALL	116	45	-	-
Topp et al. (2014) [5]	Blinatumomab	R/R B-ALL	36	32	-	-
Topp et al. (2015) [3]	Blinatumomab	B-ALL	189	39	-	-
Topp et al. (2011) [95]	Blinatumomab	B-ALL	21	47	-	-
Goebeler et al. (2016) [96]	Blinatumomab	R/R NHL	76	65	-	-
Moreau et al. (2022) [78]	Teclistamab	MM	165	64	5	100
Thieblemont et al. (2022) [21]	Epcoritamab	DLBCL, HGBCL, FL, PMBCL	157	64	3	96.8
Hutchings et al. (2021) [22]	Epcoritamab	DLBCL, FL	73	68	3	94
Linton et al. (2024) [24]	Epcoritamab	FL	214 ***	64.5 ***	2.5 ***	97.5 ***
Hutchings et al. (2021) [36]	Glofitamab	DLBCL, FL, PMBCL	171	64	3	100
Song et al. (2024) [42]	Glofitamab	DLBCL, PMBCL, HGBCL, FL	30	57.5	2	100
Budde et al. (2022) [52]	Mosunetuzumab	FL	90	60	3	100
Budde et al. (2022) [53]	Mosunetuzumab	B-NHL	197 ****	61.8 ****	3 ****	-
Bartlett et al. (2023) [56]	Mosunetuzumab	DLBCL	88	66.5	3	100
Olszewski et al. (2023) [57]	Mosunetuzumab	DLBCL	40	65	-	95
Matasar et al. (2024) [58]	Mosunetuzumab	NHL	218	64	3	100
Budde et al. (2024) [60]	Mosunetuzumab	NHL, DLBCL, HGBCL, FL	120	68	2	94.1

Legend: MM: Multiple Myeloma; B-ALL: B-cell acute lymphoblastic leukemia; B-NHL: B-Non-Hodgkin lymphoma; DLBCL: Diffuse large B-cell lymphoma; NHL: Non-Hodgkin lymphoma; R/R: Relapsed/Refractory; HSCT: Hematopoietic stem cell transplantation; HGBCL: High-grade B-cell lymphoma; Follicular Lymphoma; PMBCL: Primary mediastinal B-cell lymphoma; * = data from clinicaltrials.gov; ** = data from drug label; *** = Pivotal & Cycle 1 Optimization Cohort; **** = Aggressive NHL & Indolent NHL Cohorts.

**Table 2:** Summary table of forest plot results of cardiovascular adverse events.

Adverse Cardiac Event	Occurrence Rate (%)	95% Confidence Interval (%)	*p*-Value	I^2^ (%)
Cardiac Arrhythmias	13.6	10.1–18.2	0.05	50.1
Hypotension	13.3	10–17.3	<0.0001	76.9
Tachycardia	10.2	7.6–13.55	0.0003	64.9
Hypertension	8.0	5.8–11.0	0.1910	27.5
Sudden Death	3.4	0–80	0.0455	67.6
Acute Myocardial Infarction	1.5	0.7–3.1	0.5	0.0
Heart Failure	1.4	0.3–5.8	0.0850	48.3
Atrial Fibrillation	1.3	0.4–4.4	0.06	52.5

## Data Availability

No new data were created or analyzed in this study. Data sharing is not applicable to this article.

## Supplementary Materials

The following supporting information can be downloaded at https://www.mdpi.com/article/10.3390/pathophysiology33030060/s1. Figure S1: R analysis of acute myocardial infarction for mosunetuzumab; Figure S2: R analysis of acute myocardial infarction for glofitamab; Figure S3: R pooled analysis of acute myocardial infarction for blinatumomab, glofitamab, and mosunetuzumab; Figure S4: R analysis of cardiac arrest for blinatumomab; Figure S5: R analysis of atrial fibrillation for blinatumomab; Figure S6: R pooled analysis of atrial fibrillation for blinatumomab, mosunetuzumab, and elranatamab; Figure S7: R analysis of tachycardia for nmosunetuzumab; Figure S8: R analysis of tachycardia for elranatamab; Figure S9: R analysis of tachycardia for blinatumomab; Figure S10: R pooled analysis of tachycardia for blinatumomab, epcoritamab, glofitamab, mosunetuzumab, elranatamab, and talquetamab; Figure S11: R analysis of supraventricular tachycardia for blinatumomab; Figure S12: R analysis of bradycardia for blinatumomab; Figure S13: R analysis of cardiac arrhythmia for blinatumomab; Figure S14: R pooled analysis of cardiac arrhythmia for blinatumomab, teclistamab, epcoritamab, and elranatamab; Figure S15: R analysis of hypotension for mosunetuzumab; Figure S16: R analysis of hypotension for elranatamab; Figure S17: R analysis of hypotension for blinatumomab; Figure S18: R pooled analysis of hypotension for blinatumomab, mosunetuzumab, epcoritamab, glofitamab, teclistamab, elranatamab, and talquetamab; Figure S19: R analysis of hypertension for elranatamab; Figure S20: R analysis of hypertension for blinatumomab; Figure S21: R pooled analysis of hypertension for blinatumomab and elranatamab; Figure S22: R analysis of heart failure for blinatumomab; Figure S23: R pooled analysis of heart failure for blinatumomab and epcoritamab; Figure S24: R pooled analysis of cardiopulmonary failure for blinatumomab and elranatamab; Figure S25: R analysis of sudden death for elranatamab; Figure S26: R pooled analysis of sudden death for mosunetuzumab and elranatamab; Figure S27: R pooled analysis of cough for blinatumomab and elranatamab; Figure S28: R analysis of subclavian vein thrombosis for blinatumomab; Figure S29: Pooled analysis of adverse events of all grades for blinatumomab and elranatamab; Table S1: Proportion of adverse cardiac events in the included studies; Table S2: Baseline characteristics of the studies; Table S3: Summary of Forest plot results; File S1: AMSTAR 2: a critical appraisal tool for systematic reviews that include randomised or nonrandomized studies of healthcare interventions, or both.
