# Supplementary material for: A Comprehensive Meta-Analysis of the Cardiovascular Adverse Effects of Bispecific Antibody Therapy in Hematologic Malignancies
Source: Pathophysiology. 2026 Aug 11;33(3):60. doi: 10.3390/pathophysiology33030060 (PMC13510379; doi:10.3390/pathophysiology33030060)
Supplement: Supplementary file 1 [file pathophysiology-33-00060-s001.zip › Supplemental Data 2.pdf]

Study	Number of Patients	Cardiac Arrhythmia (%)	Tachycardia (%)	SVT (%)	Bradycardia (%)	AFib (%)	Hypertension (%)	Hypotension (%)	Heart Failure (%)	CP Failure (%)	AMI (%)	Cardiac Arrest (%)	Sudden Death (%)
Lesokhin et al. [MagnetisMM-3, Cohort A] (2023)	123	-	5.7*	-	-	0.81*	8.1*	9.8*	-	0.8*	-	-	-
Lesokhin et. al. [MagnetisMM-3, Cohort B] (2023)	64*	-	7.8*	-	-	-	7.8*	7.8*	-	-	-	-	-
Lesokhin et. al. [MagnetisMM-3, Cohorts A &B] (2023)	187*	16**	-	-	-	-	-	-	-	-	-	-	-
Bahlis et. al. [MagnetisMM-1] (2023)	55	-	-	-	-	-	-	-	-	-	-	-	1.8
Iida et. al. [MagnetisMM-2] (2024)	4	-	-		-	-	--	-	-	-	-	-	25
Rasche et al. [MonumenTAL-1 Phase 2] (2024)	339**	-	11**	-	-	-	-	21**	-	-	-	-	-
Kantarjian et al. [TOWER Study] (2017) (n discrepancy)	271	14*	6.74*	-	-	0.37*	6.4*	12	0.4	0.4*	2.2	0.37	-
Katz et al. (2022)	28	-	-	-	-	-	-	14.3*	-	-	-	-	-
Gaballa et al. (2022) (n discrepancy)	23*	-	-	-	-	-	-	4.35*	-	-	-	-	-
Rossi et al. (2021) (n discrepancy)	35*	-	-	-	-	-	2.86*	2.86*	-	-	-	-	-
Zhou et al. (2022) (n discrepancy)	121*	-	-	-	-	-	-	5.83*	-	-	-	0.83*	-
Viardot et al. [Cohort 1] (2016)	9	-	11.11*	11.11*	-	-	-	11.11*	11.11*	-	-	-	-
Viardot et al. [Cohort 2] (2016)	2	50*	-	-	-	-	-	-	-	-	-	-	-
Viardot et al. [Cohort 3] (2016)	14	-	-	-	-	-	-	-	-	-	-	-	-
Martinelli et al. (2017)	45	-	8.9*	-	-	6.7*	8.9*	13.3*	-	-	-	-	-
Kiyoi et al. [Phase 1b Cohort] (2020)	5	-	-	-	-	-	-	20*	-	-	-	-	-
Kiyoi et al. [Phase 2 Cohort] (2020)	21	-	-	-	-	-	-	4.76*	4.76*	-	-	-	-
Goto et al. (2022)	14	7.14*	-	-	-	-	7.14*	-	-	-	-	-	-
Coyle et al. (2020)	41	-	-	--	-	2.44*	-	-	-	-	-	-	-
Gokbuget et al. (2018)	116	-	-		-	-	6.0*	12.1*	-	-	-	-	-
Topp et al. (2014)	36	2.78*	25*	-	2.78*	-	22.2*	11.1*	-	-	-	-	-
Topp et al. (2015)	189	-	5.82*	0.53*	0.53*	0.53*	6.35*	11.1*	0.53*	-	-	-	-
Topp et al. (2011)	21	-	19.05*	-	9.52*	-	-	-	-	-	-	-	-
Goebeler et al. (2016)	76	1.32*	9.21*	1.32*	1.32*	-	9.21*	10.53*	1.32*	-	-	-	-
Moreau et al. (2022)	165	16**	-	-	-	-	-	18**	-	-	-	-	-
Thieblemont et al. (2022)	157	10**	-	-	-	-	-	-	-	-	-	-	-
Hutchings et al. (2021) (Epcoritamab)	73	-	21	-	-	-	-	29	-	-	-	-	-
Linton et al. (2024)	214 ‡	-	-	-	-	-	-	-	0.47 †	-	-	-	-
Hutchings et al. (2021)	171	-	15.8	-	-	-	-	24.6	-	-	0.6	-	-
Song et al. (2024)	30	-	-	-	-	-	-	-	-	-	3.3	-	-
Budde et al. (2022)	90	-	28	-	-	-	-	38	-	-	-	-	-
Budde et al. (2021)	197‡	-	4.06‡	-	-	-	-	3.55‡	-	-	-	-	-
Bartlett et al. (2023)	88	-	-	-	-	-	-	5.68	-	-	1.13	-	-
Olszewski et al. (2023)	40	-	-	-	-	-	-	25	-	-	-	-	-
Matasar et al. (2024)	218	-	9.63	-	-	-	-	13.76	-	-	0.45	-	-
Budde et al. (2023)	120	-	-	-	-	-	-	-	-	-	0.83	-	0.8

Table S1. Proportion of adverse cardiac events in the included studies.

SVT: Supraventricular tachycardia; AFib : Arterial Fibrillation; CP Failure: Cardiopulmonary Failure; AMI: Acute Myocardial Infarction; *=data from clinicaltrials.gov; **=Data from drug label; † =Pivotal & Cycle 1 Optimization Cohort; ‡: Aggressive NHL & Indolent NHL Cohorts

Study	ClinicalTrial.gov ID	Drug	Pathology	Number of Patients	Median Age (Years)	Number of prior treatments (median)	ECOG Performace Status ≤ 1 (%)
Lesokhin et al. [MagnetisMM-3, Cohort A] (2023)	NCT04649359	Elranatamab	MM	123	68	5	94.3
Lesokhin et. al. [MagnetisMM-3, Cohort B] (2023)	NCT04649359		MM	64*	65.7*	7.5*	-
Lesokhin et. al. [MagnetisMM-3, Cohorts A &B] (2023)	NCT04649359		MM	187**	68**	-	-
Bahlis et. al. [MagnetisMM-1] (2023)	NCT03269136		MM	55	64	5	90.9
Iida et. al. [MagnetisMM-2] (2024)	NCT04798586		MM	4	68.5	7.5	100
Rasche at al. [MonumenTAL-1 Phase 2] (2024)	NCT04634552	Talquetamab	MM	339**	67	5	-
Kantarjian et al. [TOWER Study] (2017)	NCT02013167	Blinatumomab	B-ALL	271	40.8	-	84.8
Katz et al. (2022)	NCT03023878		High-risk DLBCL	28	64	-	-
Gaballa et al. (2022)	NCT02807883		B-ALL	23*	29	-	-
Rossi et al. (2021)	NCT02961881		R/R Indolent NHL	35*	64	-	-
Zhou et al. (2022)	NCT03476239		B-ALL	121*	35.4*	-	80
Viardot et al. [Cohort 1] (2016)	NCT01741792		DLBCL	9	71.8*	3	-
Viardot et al. [Cohort 2] (2016)	NCT01741792		DLBCL	2	64.5*	2	-
Viardot et al. [Cohort 3] (2016)	NCT01741792		DLBCL	14	57.1	3	-
Martinelli et al. (2017)	NCT02000427		B-ALL	45	55	-	80
Kiyoi et al. [Phase 1b Cohort] (2020)	NCT02412306		R/R B-ALL	5	58	-	80
Kiyoi et al. [Phase 2 Cohort] (2020)	NCT02412306		R/R B-ALL	21	43	-	95
Goto et al. (2022)	NCT02412306		R/R B-ALL	14	-	-	-
Coyle et al. (2020)	NCT02910063		R/R B-cell NHL	41	56	-	90
Gokbuget et al. (2018)	NCT01207388		B-ALL	116	45	-	-
Topp et al. (2014)	NCT01209286		R/R B-ALL	36	32	-	-
Topp et al. (2015)	NCT01466179		B-ALL	189	39	-	-
Topp et al. (2011)	NCT00560794		B-ALL	21	47	-	-
Goebeler et al. (2016)	NCT00274742		R/R NHL	76	65	-	-
Moreau et al. (2022)	NCT03145181 & NCT04557098	Teclistamab	MM	165	64	5	100
Thieblemont et al. (2022)	NCT03625037	Epcoritamab	DLBCL, HGBCL, FL, PMBCL	157	64	3	96.8
Hutchings et al. (2021)	NCT03625037		DLBCL, FL	73	68	3	94
Linton et al. (2024)	NCT03625037		FL	214†	64.5†	2.5†	97.5†
Hutchings et al. (2021)	NCT03075696	Glofitamab	DLBCL, FL, PMBCL	171	64	3	100
Song et al. (2024)	NCT04657302		DLBCL, PMBCL, HGBCL, FL	30	57.5	2	100
Budde et al. (2022)	NCT02500407	Mosunetuzumab	FL	90	60	3	100
Budde et al. (2021)	NCT02500407		B-cell NHL	197‡	61.8‡	3‡	-
Bartlett et al. (2023)	NCT02500407		DLBCL	88	66.5	3	100
Olszewski et al. (2023)	NCT03677141		DLBCL	40	65	-	95
Budde et al. (2023)	NCT03671018		NHL, DLBCL, HGBCL, FL	120	68	2	94.6
Matasar et al. (2024)	NCT02500407		NHL	218	64	3	100

Table S2. Baseline characteristics of the studies

MM: Multiple Myeloma; B-ALL: B-cell acute lymphoblastic leukemia; DLBCL: Diffuse large B-cell lymphoma; NHL: Non-Hodgkin lymphoma; R/R: Relapsed/Refractory; HGBCL: High-grade B-cell lymphoma; Follicular Lymphoma; PMBCL: Primary mediastinal B-cell lymphoma; *=data from clinicaltrials.gov; **=Data from drug label; † =Pivotal & Cycle 1 Optimization Cohort; ‡: Aggressive NHL & Indolent NHL Cohorts

Adverse Cardiac Event	Occurrence Rate (%)	95% Confidence Interval (%)	P-Value	I^2 (%)
Cardiac Arrhythmia	13.6	10.1-18.2	0.05	50.1
Hypotension	13.3	10-17.3	<0.0001	76.9
Tachycardia	10.2	7.6-1.35	0.0003	64.9
Hypertension	8	5.8-11	0.1910	27.5
Sudden Death	3.4	0-80	0.0455	67.6
Acute Myocardial Infarction	1.5	0.7-3.1	0.5	0
Heart Failure	1.4	0.3-2.6	0.0850	48.3
Atrial Fibrillation	1.3	0.4-4.4	0.06	52.5

Table S3. Summary of Forest plot results
