# Supplementary material for: A Comprehensive Meta-Analysis of the Cardiovascular Adverse Effects of Bispecific Antibody Therapy in Hematologic Malignancies
Source: Pathophysiology. 2026 Aug 11;33(3):60. doi: 10.3390/pathophysiology33030060 (PMC13510379; doi:10.3390/pathophysiology33030060)

Supplemental Data

Supplemental Figures S1-29 describe the forest plot figures completed through R analysis on the bispecific antibodies (BsAbs) blinatumomab, elranatamab, epcoritamab, glofitamab, mosunetuzumab, talquetamab, and teclistamab. They describe acute myocardial infarction, cardiac arrest, atrial fibrillation, tachycardia, supraventricular tachycardia, bradycardia, cardiac arrhythmia, hypotension, hypertension, heart failure, cardiopulmonary failure, cough, subclavian vein thrombosis, and sudden death. Supplemental Figure S29 is a pooled analysis of all adverse events of all grades.

Supplemental Forest Plot Figures

Figure S1 R analysis of acute myocardial infarction for mosunetuzumab

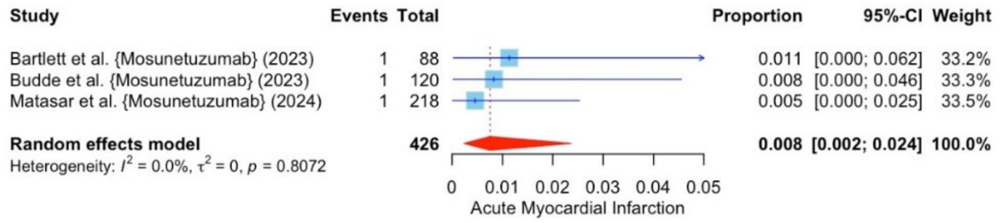

Figure S2 R analysis of acute myocardial infarction for glofitamab

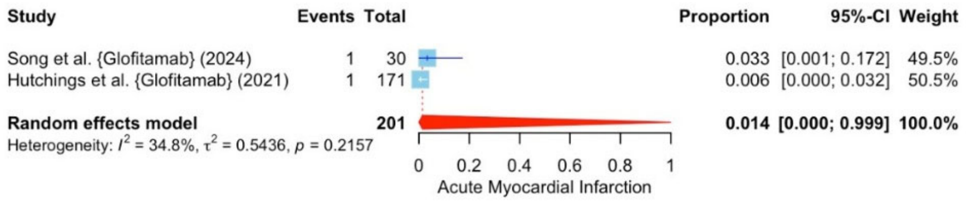

Figure S3 R pooled analysis of acute myocardial infarction for blinatumomab, glofitamab, and mosunetuzumab

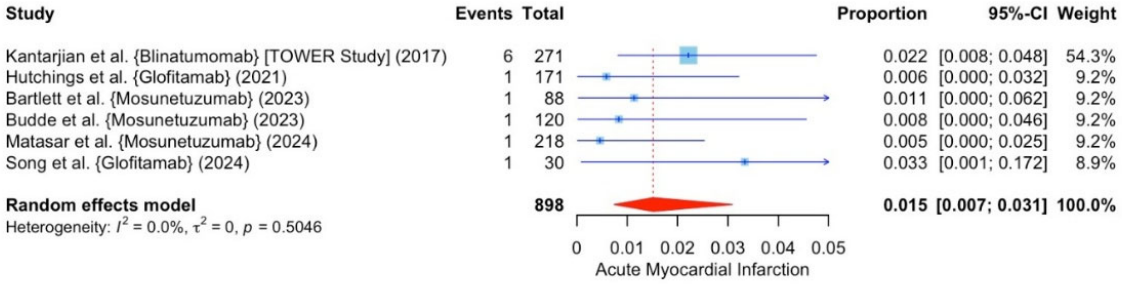

Figure S4 R analysis of cardiac arrest for blinatumomab

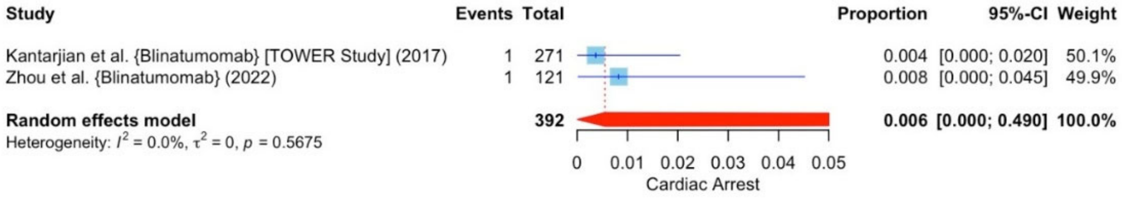

Figure S5 R analysis of atrial fibrillation for blinatumomab

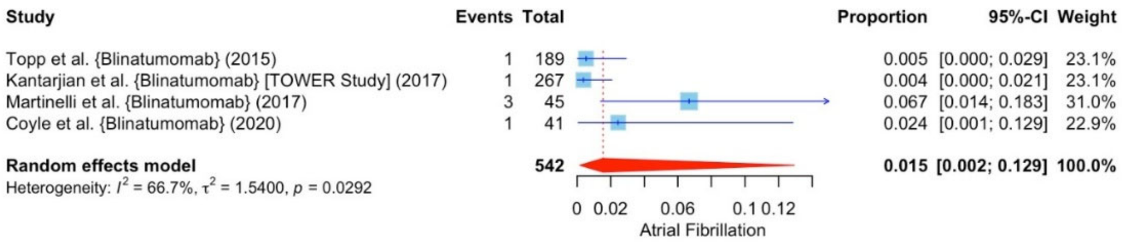

Figure S6 R pooled analysis of atrial fibrillation for blinatumomab, mosunetuzumab, and elranatamab

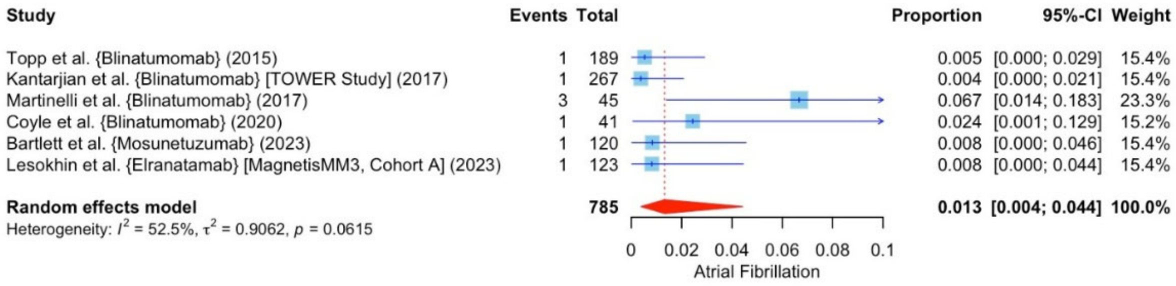

Figure S7 R analysis of tachycardia for nmosunetuzumab

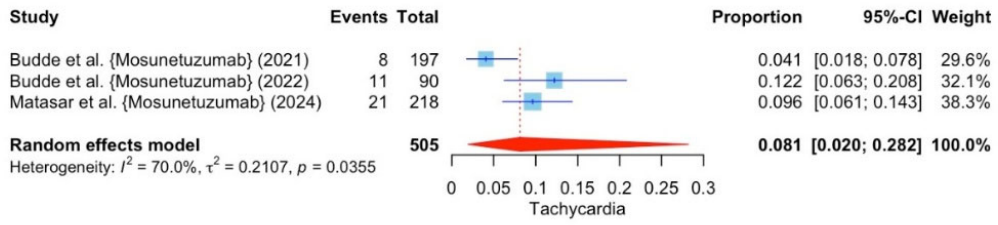

Figure S8 R analysis of tachycardia for elranatamab

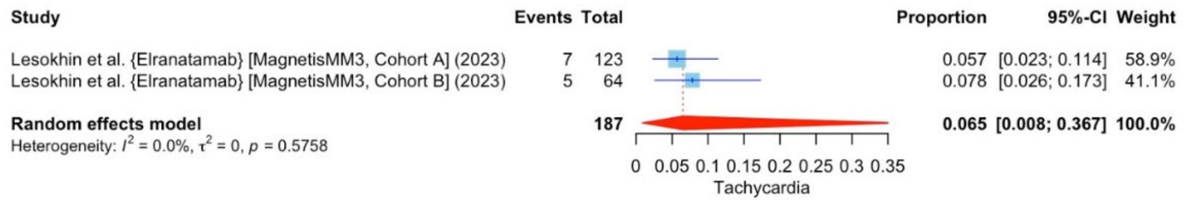

Figure S9 R analysis of tachycardia for blinatumomab

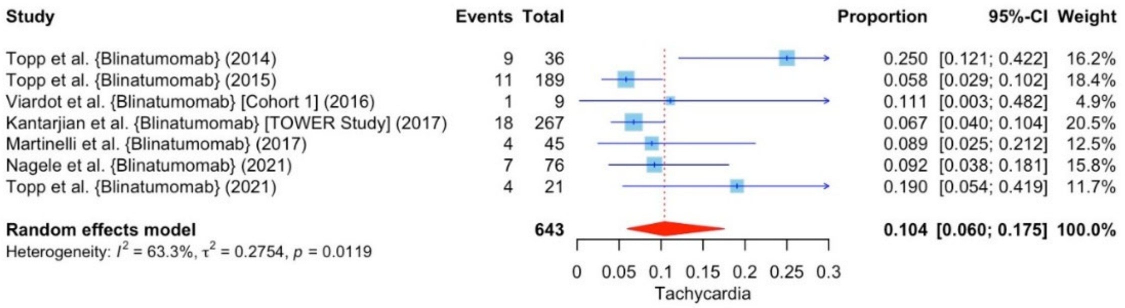

Figure S10 R pooled analysis of tachycardia for blinatumomab, epcoritamab, glofitamab, mosunetuzumab, elranatamab, and talquetamab

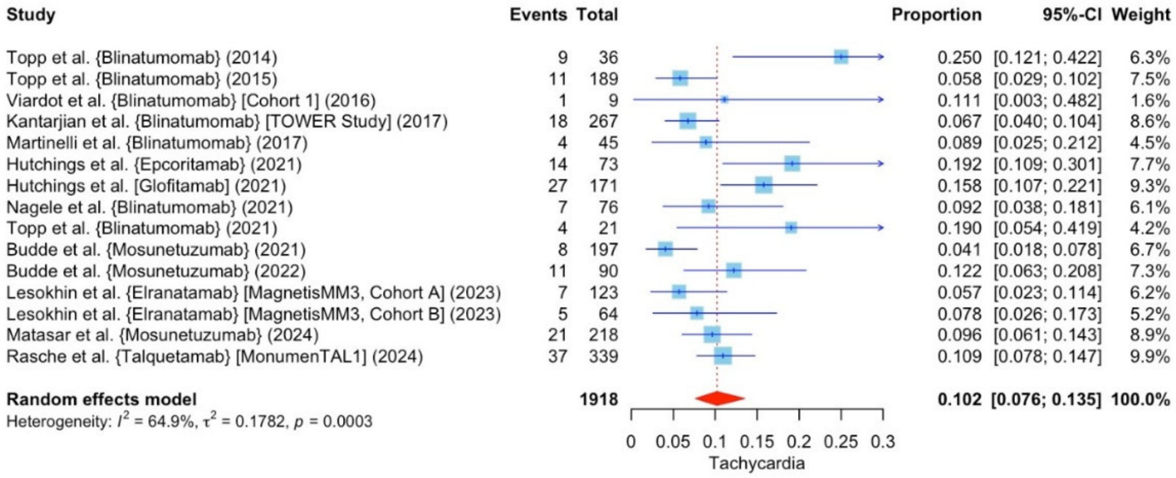

Figure S11 R analysis of supraventricular tachycardia for blinatumomab

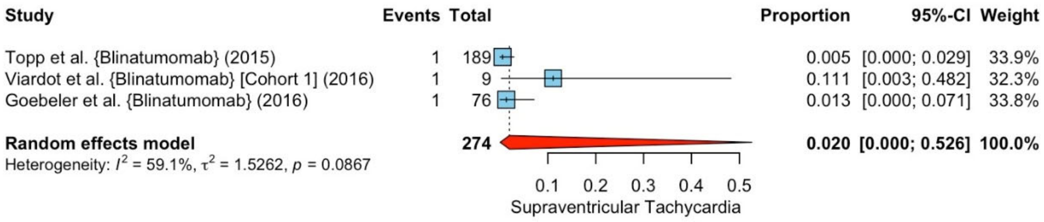

Figure S12 R analysis of bradycardia for blinatumomab

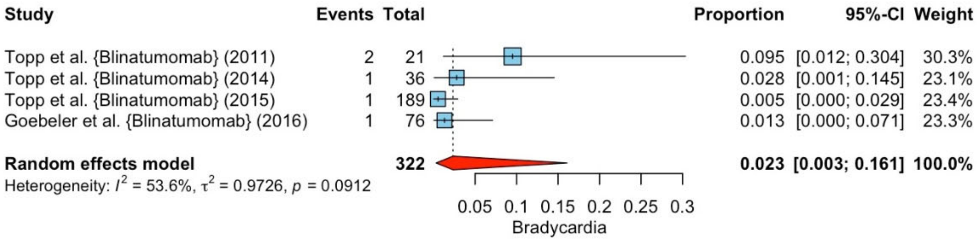

Figure S13 R analysis of cardiac arrhythmia for blinatumomab

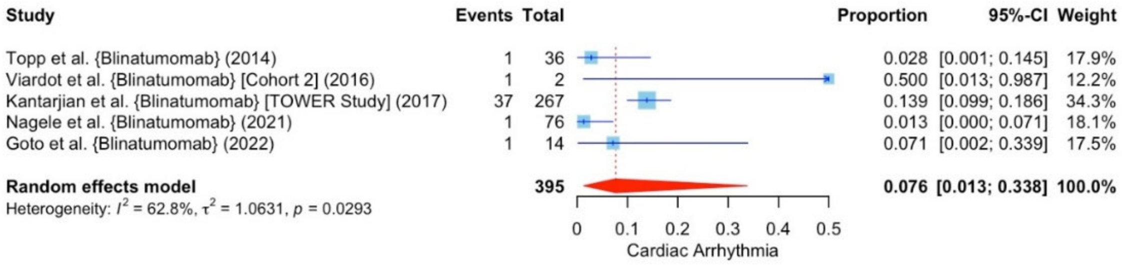

Figure S14 R pooled analysis of cardiac arrhythmia for blinatumomab, teclistamab, epcoritamab, and elranatamab

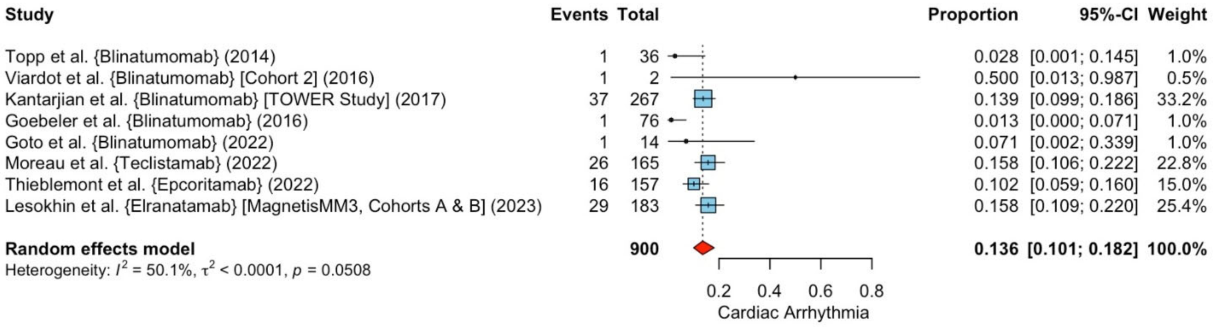

Figure S15 R analysis of hypotension for mosunetuzumab

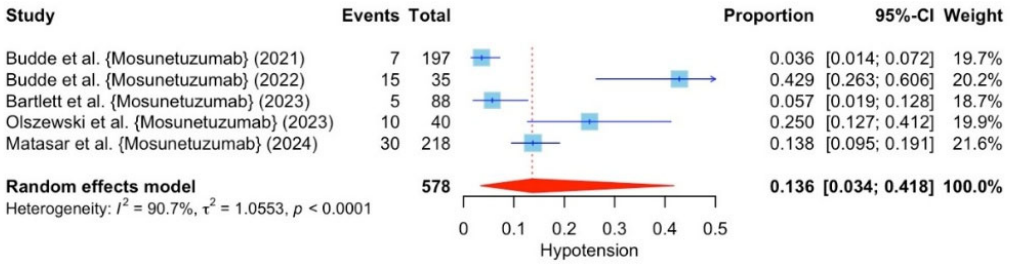

Figure S16 R analysis of hypotension for elranatamab

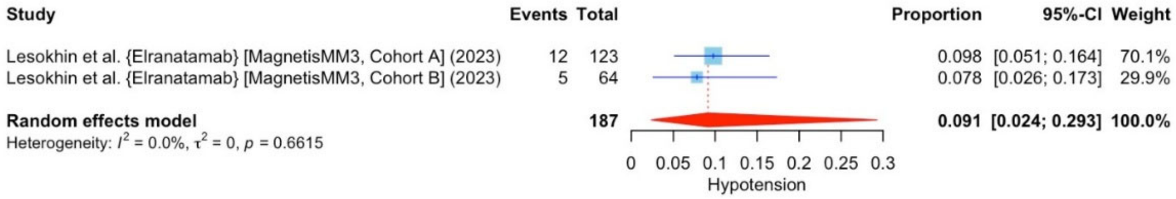

Figure S17 R analysis of hypotension for blinatumomab

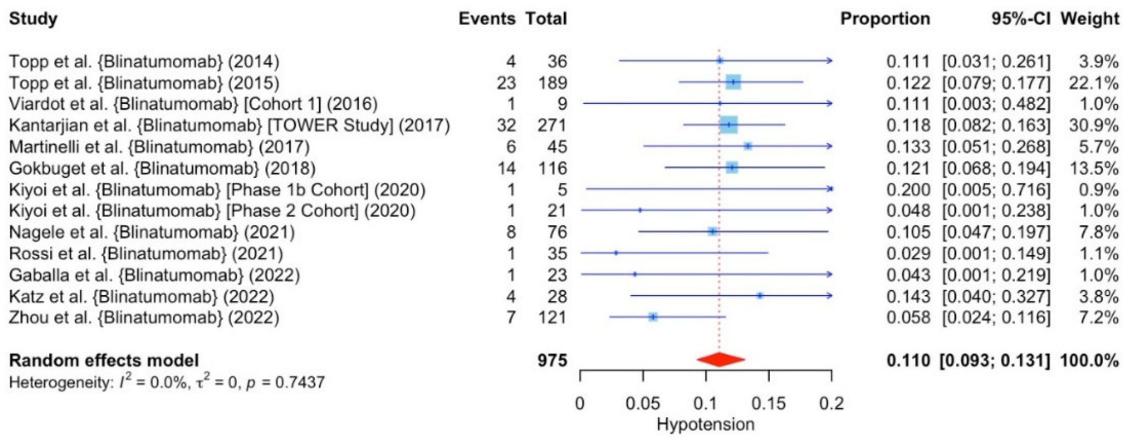

Figure S18 R pooled analysis of hypotension for blinatumomab, mosunetuzumab, epcoritamab, glofitamab, teclistamab, elranatamab, and talquetamab

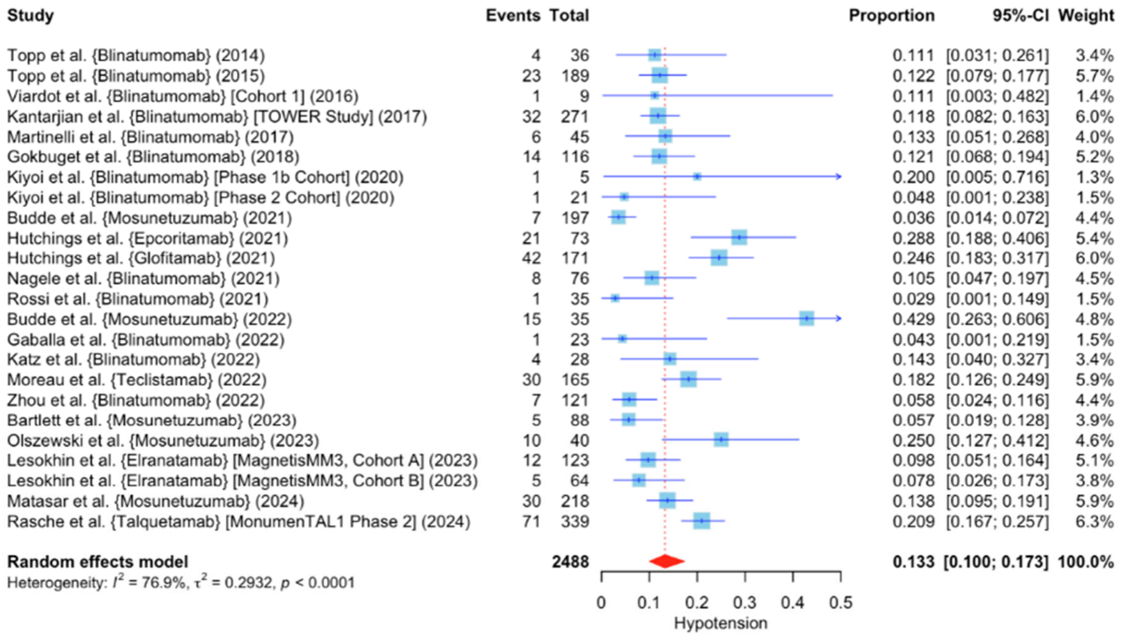

Figure S19 R analysis of hypertension for elranatamab

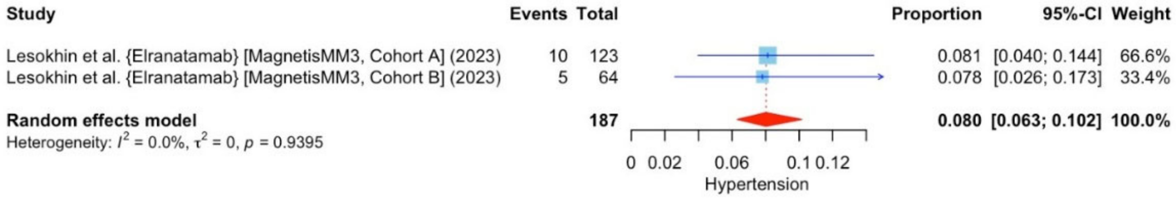

Figure S20 R analysis of hypertension for blinatumomab

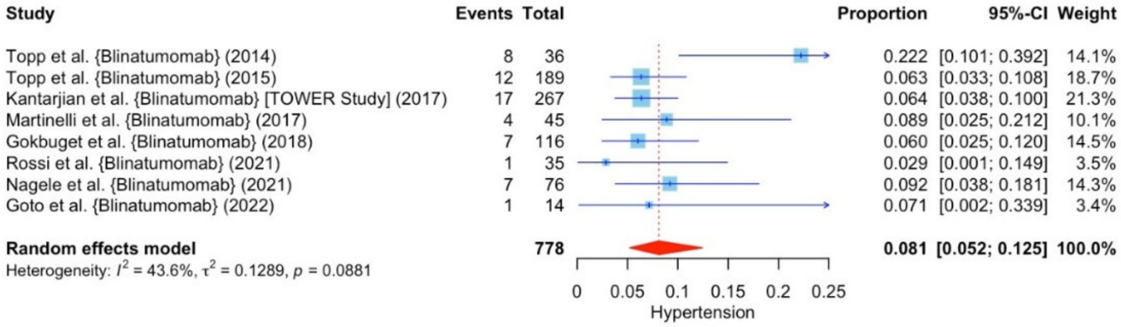

Figure S21 R pooled analysis of hypertension for blinatumomab and elranatamab

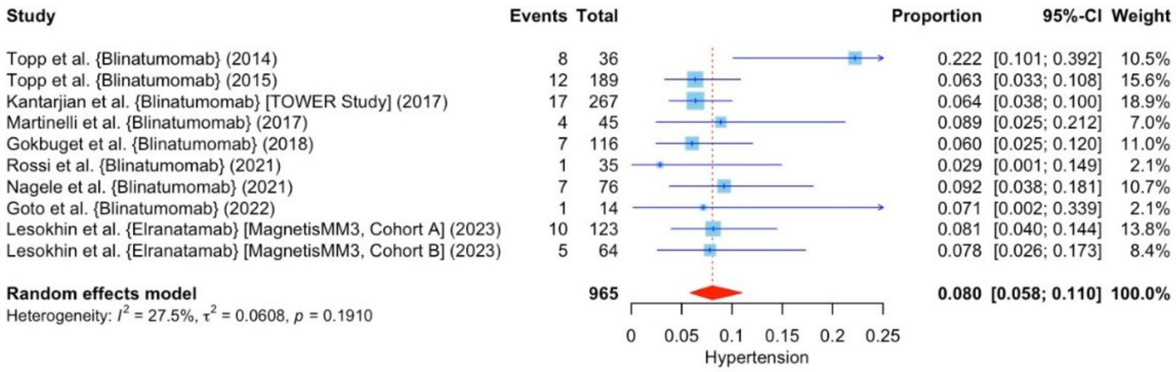

Figure S22 R analysis of heart failure for blinatumomab

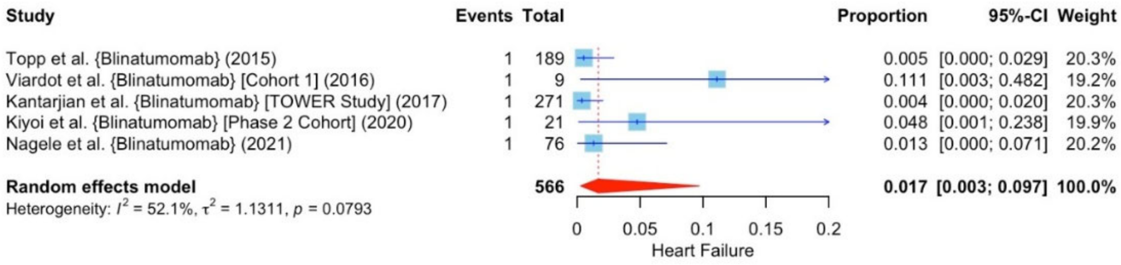

Figure S23 R pooled analysis of heart failure for blinatumomab and epcoritamab

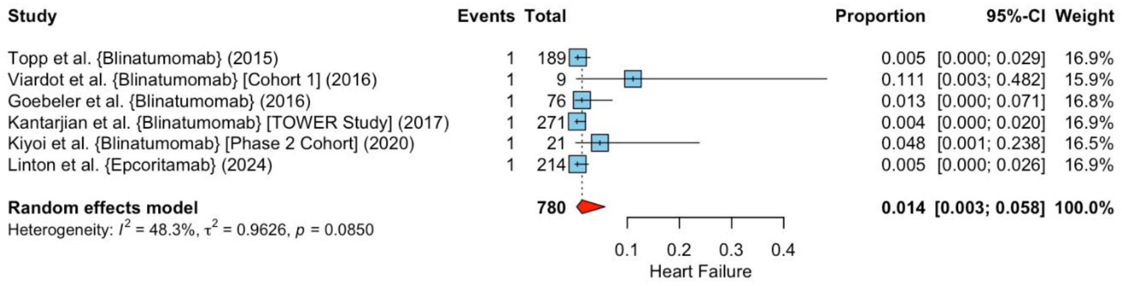

Figure S24 R pooled analysis of cardiopulmonary failure for blinatumomab and elranatamab

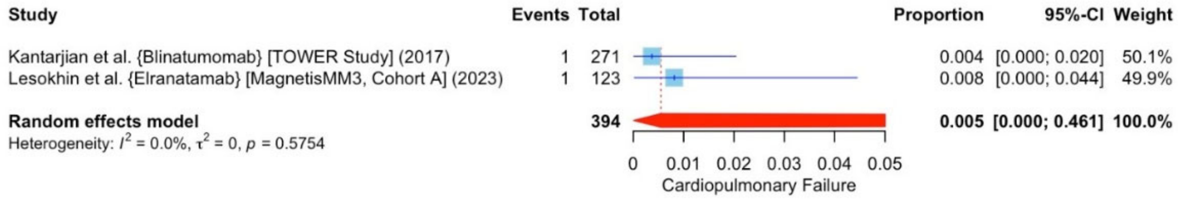

Figure S25 R analysis of sudden death for elranatamab

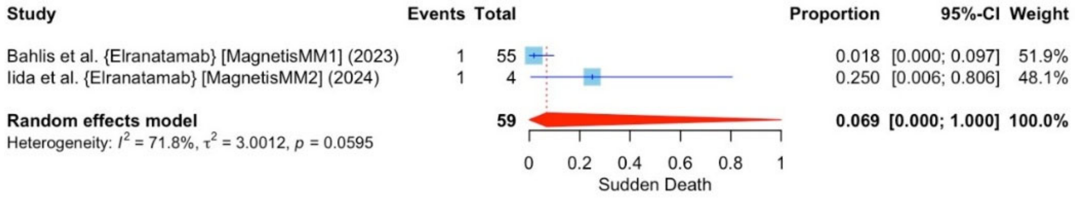

Figure S26 R pooled analysis of sudden death for mosunetuzumab and elranatamab

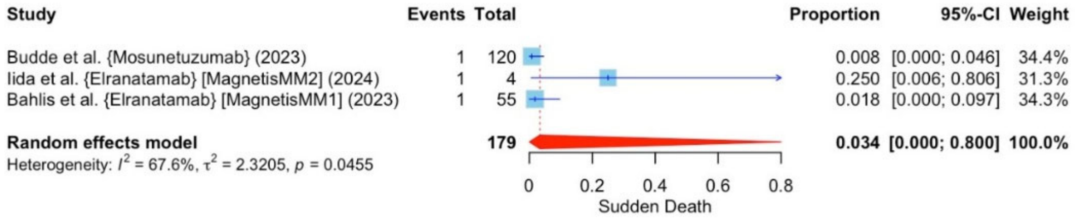

Figure S27 R pooled analysis of cough for blinatumomab and elranatamab

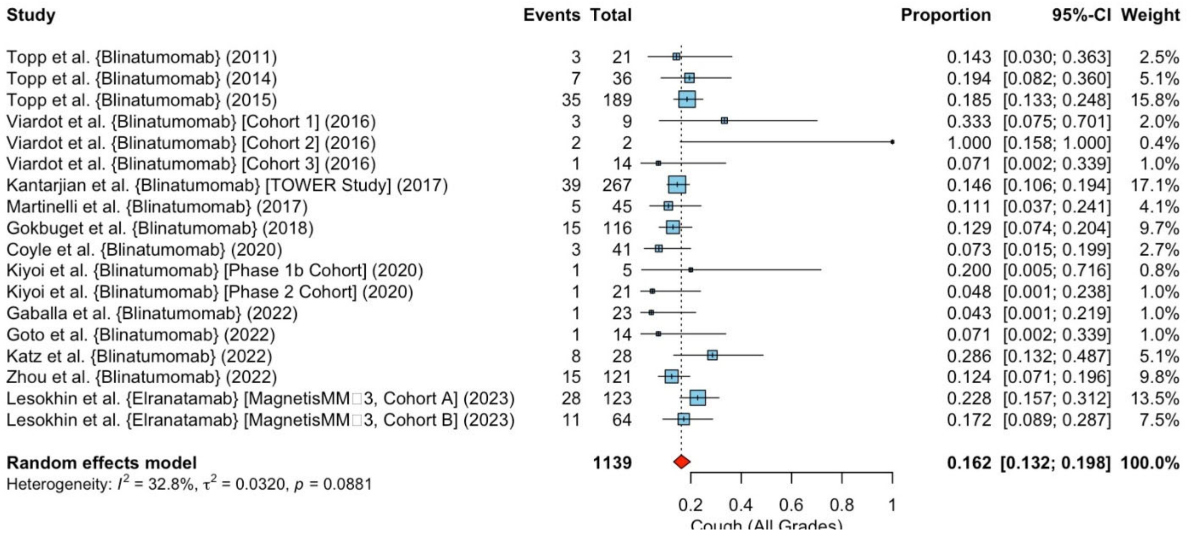

Figure S28 R analysis of subclavian vein thrombosis for blinatumomab

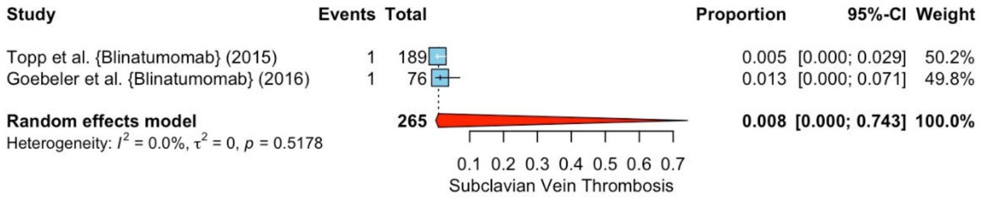

Figure S29 Pooled analysis of adverse events of all grades for blinatumomab and elranatamab

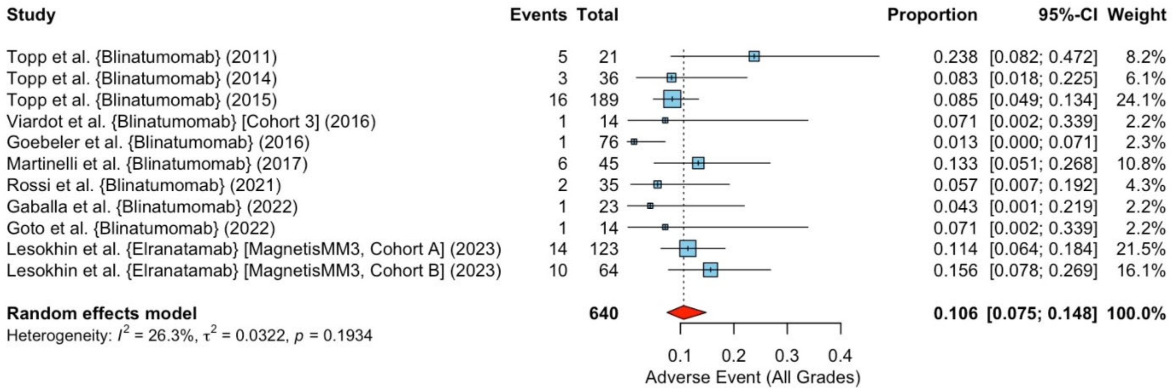

Supplement: Supplementary file 1 [file pathophysiology-33-00060-s001.zip › Supplemental Data.pdf]
